# Development of maximum power point tracking algorithm based on Improved Optimized Adaptive Differential Conductance Technique for renewable energy generation

**DOI:** 10.1016/j.heliyon.2024.e41344

**Published:** 2024-12-21

**Authors:** Val Hyginus Udoka Eze, Martin Chinweokwu Eze, Samuel A. Ugwu, Valentine S. Enyi, Wisdom O. Okafor, Chibuzo C. Ogbonna, Ogbonna U. Oparaku

**Affiliations:** a Department of Publication and Extension Unit, Kampala International University, Uganda; b Department of Electrical, Telecommunication and Computer Engineering, Kampala International University, Uganda; c School of Engineering, Cardiff University, Cardiff, CF24 3AA, United Kingdom; d Department of Electronic Engineering, University of Nigeria, Nsukka, 410001, Enugu, Nigeria

## Abstract

Maximum Power Point Tracking (MPPT) is a technique employed in photovoltaic (PV) systems to ensure that the modules transfer the maximum generated power to the load. An advanced algorithm, the Improved Optimized Adaptive Differential Conductance (IOADC), was developed by applying Kirchhoff's law within a single diode model framework. The algorithm's performance was evaluated under various solar irradiance levels of 500 W/m^2^, 750 W/m^2^, and 1000 W/m^2^ at a constant temperature of 298K, analyzing its impact on power generation and transfer. Additionally, the performance was assessed at varying temperatures of 250K, 298K, and 350K under a constant irradiance of 1000 W/m^2^ to examine its effect on the Module Saturation Current (MSC). The analysis revealed that the PV modules' impedance decreases with increasing irradiance, while the load's impedance remains largely unaffected which aligns with the PV applications. However, the implementation of the IOADC technique showed significant effectiveness. It was also noted that an increase in temperature raises the module saturation current, which in turn reduces the power output, and vice versa which also agrees with the PV application. Real-world application results indicated that at an irradiance of 750 W/m^2^, the output power at the maximum power point (MPP) for the Optimized Adaptive Differential Conductance (OADC), Voltage Control Technique, and IOADC were 83.3346 W, 86.9122 W, and 100.1739 W, respectively. The 100.1739W obtained from the IOADC technique showed a significant improvement. Through comprehensive comparative evaluation, analysis, and validation of the effects of varying temperature, irradiance, and MSC on output power, the developed IOADC model demonstrated a relative improvement of 15.82 % in simulations and 20.21 % in real-world conditions compared to the Voltage Control Technique and the OADC technique, respectively. Simulation validation and real-world application validation were performed using MATLAB 2020b. These validations confirmed the superior performance of the IOADC algorithm under varying conditions of temperature, irradiance, and module saturation current.

## 1 Introduction

The swift surge in contemporary lifestyles, population growth, urbanization, and rapid industrial expansion has significantly heightened the need for energy, placing substantial pressure on the energy sector. This increased demand has led to the depletion of traditional energy sources such as fossil fuels, resulting in elevated energy costs, insufficient energy generation, and a constrained energy supply. Furthermore, urbanization and population growth have contributed to the continuous emission of nitrogen oxide (NO_2_), ammonia (NH_3_), mercury (Hg), and carbon dioxide (CO_2_) from combustion vehicles and industries, adversely affecting human health [1,2]. The release of these harmful substances intensifies climate change by enhancing the greenhouse effect, trapping heat in the atmosphere, and leading to global warming. Air pollution from fossil fuel combustion releases other harmful pollutants, such as particulate matter, nitrogen oxides, and volatile organic compounds, which have detrimental effects on respiratory and cardiovascular health [[3], [4], [5], [6]].

Addressing the ever-growing global energy needs, renewable energy has captivated the attention of governments, researchers, and energy policymakers. This fascination is attributed to the inherent abundance and user-friendly nature of renewable sources. Seen as pivotal in meeting the escalating demand for power globally, renewable energy sources are noted for their plentiful availability, environmental sustainability, and cost-effectiveness in maintenance. Solar energy, wind energy, tidal energy, biomass, and geothermal energy are noteworthy examples, each contributing to a sustainable energy landscape. Among these, wind and solar energy stand out as frontrunners due to their pollution-free nature, inexhaustibility, and abundant availability, positioning themselves at the forefront of the renewable energy revolution. Solar energy can be harnessed and transformed into electrical power through solar thermal and solar photovoltaic techniques, representing significant advancements in sustainable energy conversion [4,7].

A comprehensive analysis of global energy consumption by the International Energy Agency (IEA) forecasts that by 2050, over 45 % of the world's energy demand will be met exclusively by PV systems [8]. The fundamental building block of a photovoltaic system is the PV module, composed of solar cells. These solar photovoltaic cells, crafted from semiconductor materials like silicon, gallium arsenide, and cadmium telluride, have the unique capability to directly convert sunlight into electricity [9]. The power conversion efficiency (PCE) of a solar module is predominantly influenced by operating temperature and irradiance, marking key determinants in advancing solar energy technologies [[10], [11], [12], [13], [14]].

Currently, scientists across diverse disciplines are fervently investigating the designs, constructions, and control mechanisms of photovoltaic systems to address the impending surge in energy demand. This surge is intricately linked to the mechatronic essence of solar photovoltaic panels [15]. The solar photovoltaic (PV) technique stands out as the most pragmatic means of harnessing electricity from sunlight through PV cells [16,17]. Solar PV has emerged as a widely embraced renewable energy source with the inherent benefits of low operational costs, environmental sustainability, and minimal maintenance requirements [18].

Photovoltaic systems are evaluated based on their maximum power, which represents the highest attainable power output under Standard Test Conditions (STC) where solar irradiance is 1000 W/m^2^, temperature is 25 °C, and air mass is 1.5 [19]. To effectively and efficiently harness and transfer the generated power to the load, the integration of a Maximum Power Point Tracker (MPPT) is crucial. The optimal performance of PV modules occurs when the PV impedance matches the load impedance. A primary factor contributing to impedance mismatch in solar photovoltaic systems is Partial Shading Condition (PSC), which causes the PV array to display multiple peaks in its output curve, leading to power losses and decreased overall efficiency [[20], [21], [22], [23]].

Research scholars in Refs. [7,[24], [25], [26], [27], [28], [29]] investigated various MPPT techniques based on their suitability for meeting industrial demands and purposes. The selection of a specific technique for modification in the context of solar PV MPPT depends on the researcher's goals and objectives. MPPT techniques are broadly categorized into intelligent and non-intelligent methods [30]. The scholars in Ref. [31] classified modern MPPT algorithms for Wind Energy Conversion Systems (WECSs) into conventional, intelligent, and hybrid. This study provides an overview of these modern MPPT algorithms as applied to permanent magnet synchronous generators in WECSs, focusing on methods based on speed convergence, efficiency, self-training, complexity, and the measurement of wind parameters.

Many researchers have explored innovative intelligent MPPT techniques, investigating various ways in which artificial intelligence can optimize power extraction in solar photovoltaic systems [32,33]. For instance, the application of sophisticated soft computing and machine learning methodologies to optimize the extraction of maximum power from solar PV systems has been discussed extensively in the literature [32,[34], [35], [36]].

Intelligent-based MPPT techniques, such as those utilizing machine learning or advanced algorithms, can more accurately track the optimal operating point of solar PV systems compared to traditional non-intelligent methods. These intelligent techniques, however, come with several drawbacks, including increased circuit complexity, higher costs, longer response times, and reduced reliability. These limitations impact their robustness and practical application in real-world scenarios. Due to these drawbacks, this research delves into exploring traditional non-intelligent MPPT techniques.

Examples of non-intelligent MPPT techniques include Perturb and Observe (P&O), Incremental Conductance (IC), Differential Conductance (DC), and Optimized Adaptive Differential Conductance (OADC). OADC, as one example of a non-intelligent MPPT model, operates by balancing the instantaneous conductance (panel impedance) and load impedance to identify the maximum operating point of solar PV [6,[30], [31], [32], [33],^37^,^38^]. This research focuses on modifying and enhancing the OADC technique due to its superior performance and robustness compared to other non-intelligent (traditional) methods. The conductance in the OADC technique is determined by the instantaneous panel conductance ($\frac{I_{\text{mpp}}}{V_{\text{mpp}}}$) and load conductance $\frac{\text{dI}}{\text{dV}}$, as described in Equation (1), where ϒ represents the resultant conductance. For Equation (1) to be ideally satisfied, the resultant conductance must be zero [36].

(1) Υ=(ImppVmpp‐dIdV)=(αnKTqRsloge(1+11000Io)[1+ki(T‐Tref)]GGref)‐I0[(qVmppαnKT)exp]Vmpp‐Ioq⍺nKTexp(VqαnKT)

The review has identified several critical challenges associated with the existing Optimized Adaptive Differential Conductance technique that affect its efficiency in tracking the Maximum Power Point (MPP) and delivering consistent power to the load. The Key identified drawbacks of OADC are (1) The OADC technique struggles with temperature variations that influence the amount of power supplied to the load. These temperature-induced fluctuations create an unstable power output, making it difficult to maintain optimal performance. (2) A significant computational error in the OADC algorithm which is the assignment of a constant value to the module saturation current (Io). Io is inherently temperature-dependent and varies with atmospheric changes. This oversight in the algorithm computation results in inaccurate power conversion, especially under partial shading conditions where temperature differences are more pronounced.

Summary of the Novelty Contributions and Innovations in the Proposed Research

➢ **Temperature Compensation Mechanisms**: This research aims to develop an Improved Optimized Adaptive Differential Conductance (IOADC) model to enhance the Maximum Power Point (MPP) tracking of solar PV systems. The key innovation is incorporating real-time temperature compensation within the IOADC algorithm. By dynamically adjusting temperature parameters based on real-time readings, the system can maintain a more stable power supply, mitigating the adverse effects of temperature fluctuations on power output. Comparative analyses will be conducted with existing voltage control techniques and OADC [37], evaluating the performance improvements of the IOADC model to draw conclusive insights.
➢ **Dynamic Saturation Current Adjustment**: The proposed IOADC model will include a dynamic, temperature-dependent calculation of the module saturation current (Io). This adjustment, accounting for temperature and atmospheric variations, is designed to enhance the accuracy of the power conversion process by ensuring precise MPP tracking under varying environmental conditions. This modification aims to significantly improve the power conversion efficiency and, consequently, the load power. The efficacy of these enhancements will be validated through comparative assessments with the conventional OADC model.
➢ **Integration of Partial Shading Handling**: The modified algorithm will be capable of detecting and adapting to partial shading conditions, thereby significantly improving system performance. This capability may involve the integration of advanced pattern recognition techniques. The effectiveness of the algorithm in handling partial shading will be rigorously tested under different irradiance levels at constant temperature and varying temperatures at constant irradiance, demonstrating the robustness and adaptability of the proposed IOADC model.

By addressing these critical areas, the proposed research seeks to advance the state-of-the-art in photovoltaic system optimization, offering practical solutions to enhance the reliability and efficiency of solar power generation.

This paper is structured into five main sections. First, the introduction to the PV system is presented, followed by the proposed methodology and mathematical derivations. Next, the results and discussions, and finally, the paper concludes with a summary and recommendation.

## 2 Proposed Methodology and Mathematical Derivation Steps

**Fig. 1:**
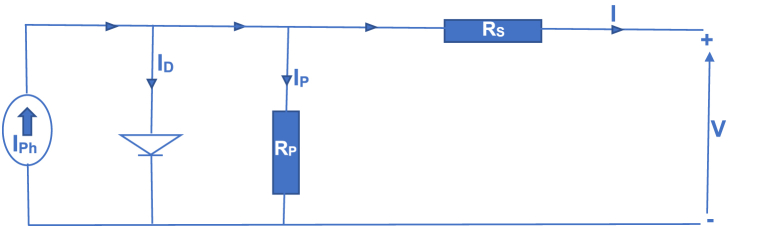
The equivalent circuit of a PV cell with a single diode [39].

The Improved Optimized Adaptive Differential Conductance Technique represents a significant advancement, utilizing a Single Diode Model (SDM) for its development. This cutting-edge approach enhances power efficiency by refining the traditional differential conductance technique within the single-diode circuit (SDC). The SDC comprises shunt resistance (Rp) and series internal resistance (Rs), as illustrated in Figure 1. The IOADC automatically adjusts certain panel parameters to ensure impedance matching, thereby optimizing power efficiency. Additionally, real-time temperature compensation parameters are incorporated into the IOADC model to address variations caused by changes in irradiance and temperature.

The development and computation of the model involve several key steps. These include the mathematical optimization of the open-circuit voltage (V_oc_) and short-circuit current (I_sc_) algorithms, the mathematical modification of the Current at Maximum Power Point (I_mpp_) and Voltage at Maximum Power Point (V_mpp_) algorithms, and the establishment of a comprehensive slope algorithm that serves as a compensation parameter. The current output algorithm will be created by the combination of all these modified algorithms. Collectively, these steps contribute to the refinement and efficacy of the IOADC Technique.

### 2.1 Mathematical Modeling of the PV Module

A photovoltaic array consists of multiple PV cells connected in series and parallel configurations. The series connections increase the voltage of the module, while the parallel connections increase the current of the cell array. In the circuit, there are resistances connected both in series (R_s_) and parallel (R_p_). An ideal solar cell is modeled by a current source in parallel with a diode, which represents the diode current and dark current [[40], [41], [42]]. Parallel resistance (R_p_) is included in the circuit, as illustrated in Figure 1, to account for dissipative phenomena and limit cell performance due to internal losses. A very high value of R_p_ significantly reduces the dark current. The shunt resistance addresses recombination losses, which are primarily due to factors such as thickness, surface area effects, and the non-ideality of the junction [43]. The single-diode equivalent electrical circuit of a solar cell consists of the photocurrent (I_ph_), the diode current (I_D_), and the dark current (I_p_), as depicted in Figure 1.

Applying and analyzing Kirchhoff's law to the nodes of the circuit shown in Figure 1 yields Equation (2).

(2) $$I=I_{\text{ph}}-I_{D}-I_{p}$$

Where: I is Output Current; $I_{\text{ph}}$ is Photo generated Current; $I_{D}$ is Diode Current and $I_{p}\;\text{is}$ dark current. When Kirchhoff’s law is applied in nodes of Figure 1, Equations (3), (4), (5), (6), (7), (8) were mathematically obtained.

(3) $$I_{\text{ph}}=I_{\text{sc}}\left[1+k_{i}\left(T-T_{\text{ref}}\right)\right]\frac{G}{G_{\text{ref}}}$$

Mathematically analyzing equation (3)

(4) $$at\;STC;\;I_{\text{ph}}=I_{\text{sc}}$$

(5) $$I_{D}=I_{o}\left(\exp\left(\frac{q\left(V+IR_{s}\right)}{\alpha \text{nkT}}\right)-1\right)$$

(6) Io=Irs[TTref]3exp[(qEgapkq)(1Tref‐1T)]

Mathematically analyzing equation (6) at STC;

(7) $$I_{o\;}=I_{\text{rs}}$$

(8) $$I_{p}=\frac{V_{D}}{R_{p}}=\frac{V+IR_{s}}{R_{p}}$$

Where; $I_{\text{sc}}$ is the short-circuit current at reference conditions; k_i_ is the temperature coefficient of the short-circuit current; T is the working cell Temperature (K); T_ref_ is reference temperature (298K); G is the working irradiance (W/m^2^); G_ref_ is the working irradiance (1000W/m^2^); I_o_ is Diode/module saturation current; q is Electron charge (1.602 x 110^-19^C); V is the voltage of the cell; k is Boltzmann’s constant (1.3865 x 10^-23^J/K); ⍺ is Diode ideality factor (0≤⍺≤2); n is the number of PV cells in series; $R_{s}$ is Resistance in series; $I_{\text{rs}}$ is the reverse saturation current; E_gap_ is the Energy bandgap of the semiconductor material (E_gap_ for silicon polycrystalline =1.1ev).

In Equation (5), a computational error in the OADC is highlighted, where the exponential component fails to encompass the constant value of -1. Notably, in Equation (6), a Dynamic Saturation Current Adjustment (DSCA) algorithm was introduced and implemented, instead of assigning a specific constant value, as exemplified in the OADC.

The PV characteristic equation was derived by substituting Equations (5), (8) into Equation (2), resulting in Equation (9). This equation differs from the output current of the OADC as observed in [37].

(9) $$I=I_{\text{ph}}-I_{o}\left(\exp\left(\frac{q\left(V+IR_{s}\right)}{\alpha \text{nkT}}\right)-1\right)-\frac{V+IR_{s}}{R_{p}}$$

Equation (9) is a general I-V characteristic equation of a single diode model [38] [[39], [44], [45], [46]]

### 2.2 Parametric Mathematical Assumptions and Approximations Made in this Research

In the course of this research, equations (9), (10), (11), (13), (14), (15), (16), (17), (19) represent the fundamental mathematical formulations that were systematically and ethically adapted to facilitate the effective development of the envisioned model. Equations (20), (21) are algorithms that directly contributed to the development of the proposed model, outlined in equation (23). Specifically, equations (20), (21) were the mathematically developed voltage and current at the maximum power point algorithms for voltage and current compensation during atmospheric change respectively. Equation (23) details the proposed Improved Optimized Adaptive Differential Conductance model developed in this research. Here are the ethical and mathematical assumptions, as well as the specified conditions, that guided the derivation of the developed algorithms.

Case 1 Given that R_p_ is very large and Rs is very small

Using the SDM for an n-cell photovoltaic system with a very large Rp and a very small Rs, Ip will tend to zero. Consequently, equation (9) can be rewritten as equation (10) [47]

(10) $$I=I_{\text{ph}}-I_{o}\left(\exp\left(\frac{q\left(V+IR_{s}\right)}{⍺\text{nKT}}\right)-1\right)$$

Since Rs is very negligible, IRs tend to zero as expressed in equation (10)

Case 2 Given that I = 0 and V = Voc

**Fig. 2:**
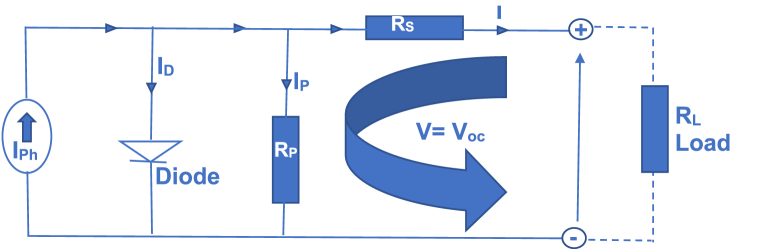
Derivation of open-circuit voltage (V_oc_) from a single diode model.

Utilizing the SDM framework depicted in Figure 2, at an open circuit, the current (I) equals zero and the voltage (V) equals the open-circuit voltage (Voc). Consequently, equation (10) transforms into equation (11).

(11) $$I_{\text{ph}}=I_{o}\left(\exp\left(\frac{qV_{\text{oc}}}{⍺\text{nKT}}\right)-1\right)$$

To obtain V_oc_, equation (11) is rewritten as in equation (12)

(12) $$V_{\text{oc}}=\frac{\alpha \text{nKT}}{q}\left(\log_{e}\left[\frac{\text{Iph}\;}{I_{o}}+1\right]\right)$$

Substituting equations (3) and (6) in equation (12), the Open-Circuit Voltage (V_oc_) is rewritten as in equation (13)

(13) $$V_{\text{oc}}=\frac{⍺\text{nKT}}{q}\left(\log_{e}\left[\frac{I_{\text{sc}}\left[1+k_{i}\left(T-T_{\text{ref}}\right)\right]\frac{G}{G_{\text{ref}}}\;}{I_{\text{rs}}\;{\left[\frac{T}{T_{ref}}\right]}^{3}\;\exp\left[\left(\frac{qE_{gap}}{\alpha k}\right)\left(\frac{1}{T_{ref}}-\frac{1}{T}\right)\right]}+1\right]\right)$$

Case 3 Given that V = 0 and I = I_sc_

**Fig. 3:**
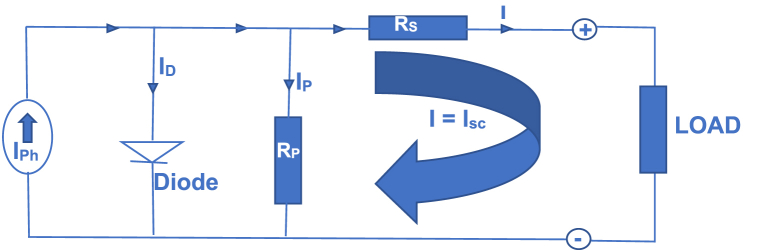
Derivation of Short Circuit Current ($I_{\text{sc}}$) from a single-diode model.

Utilizing the Single Diode Model (SDM) as depicted in Figure 3 and applying case 1, which posits that when parallel connected resistors (Rp) are significantly large, serially connected resistor (Rs) will proportionately diminish, resulting in the derivation of equation (14).

When Rp is substantially large, it indicates that the parallel resistance predominates in the electrical behavior, typically linked with the electrical resistance of cell contacts or similar elements in parallel. Conversely, a very small Rs implies that the series resistance is inconsequential compared to other elements in the model, such as the internal resistance of solar cell materials.

The decrease in the dark current (Ip) towards zero signifies the reduction of current flow through a solar cell in the absence of light, representing leakage current and the non-ideal behavior of the cell. This observation suggests that under the specified conditions (Rp very large, Rs very small), the dark current Ip tends towards zero.

Conclusively, the convergence towards zero of the dark current Ip under the specified conditions (Rp very large, Rs very small) indicates that when Rp dominates and Rs is negligible, the dark current in the photovoltaic cell modeled by SDM approaches zero. This implies an idealized scenario where the cell's electrical behavior approximates that of an ideal diode, particularly in darkness. The practical significance lies in the optimization of series and parallel resistances, which enhances the overall performance of photovoltaic cells, particularly under varying temperatures. These conditions align with the findings of Cuce et al [48].

(14) $$I_{o}\left(\exp\left(\frac{qI_{\text{sc}}R_{s}}{⍺\text{nKT}}\right)-1\right)=\left[\frac{{I_{sc}R}_{s}}{R_{p}}\right]$$

To obtain a very high Fill Factor (FF), $\frac{R_{p}}{R_{s}}\to \infty$ and $\frac{{I_{sc}R}_{s}}{R_{p}}\to 0$. However, FF is not always very high in practice, and assuming that $\frac{{I_{sc}R}_{s}}{R_{p}}\leq 10^{-3}A$, Equation (14) can be rewritten as in equation (15).

(15) $$\frac{qI_{\text{sc}}R_{s}}{⍺\text{nKT}}=\text{loge}\left(\frac{1\;}{1000I_{o}}+1\right)$$

Substitute equations (6) in equation (15) and a Short Circuit Current is obtained as in equation (16).

(16) $$I_{\text{sc}}=\frac{⍺\text{nKT}}{q}\;\log_{e}\left(\frac{{\;}_{1\;}}{1000\;\left(I_{\text{rs}}\;{\left[\frac{T}{T_{\text{ref}}}\right]}^{3}\;\exp\left[\left(\frac{qE_{\text{gap}}}{{\;}_{\alpha }k}\right)\left(\frac{1}{T_{\text{ref}}}‐\frac{1}{T}\right)\right]\right)}+1\right)$$

The output current of the PV panel is mathematically developed by substituting equations (16), (3) in equation (10) to yield equation (17).

(17) I=⍺nKTqloge(11000(Irs[TTref]3exp[(qEgapαk)(1Tref−1T)])+1)[1+ki(T−Tref)]GGref−(Irs[TTref]3exp[(qEgapαk)(1Tref−1T)])[exp(qV⍺nKT)−1]

The power delivered to the load by the PV system is given by equation (18).

(18) P=IV=(⍺nKTqloge(11000(Irs[TTref]3exp[(qEgapαk)(1Tref−1T)])+1)[1+ki(T−Tref)]GGref)∗V−((Irs[TTref]3exp[(qEgapαk)(1Tref−1T)])[exp(qV⍺nKT)−1])∗V

Where the input parameter V is such that $V_{\text{oc}}$ ≥ V ≥ 0

Therefore, differentiating equation (18) with respect to voltage (V) yield equation (19).

(19) $$\frac{\text{dP}}{\text{dV}}=\frac{⍺\text{nKT}}{q}\;\log_{e}\left(1+\frac{{\;}_{1\;}}{1000\;\left(I_{\text{rs}}\;{\left[\frac{T}{T_{\text{ref}}}\right]}^{3}\;\exp\left[\left(\frac{qE_{\text{gap}}}{{\;}_{\alpha }k}\right)\left(\frac{1}{T_{\text{ref}}}-\frac{1}{T}\right)\right]\right)}\right)\left[1+k_{i}\left(T-T_{\text{ref}}\right)\right]\frac{G}{G_{\text{ref}}}-\left(I_{\text{rs}}\;{\left[\frac{T}{T_{\text{ref}}}\right]}^{3}\;\exp\left[\left(\frac{qE_{\text{gap}}}{{\;}_{\alpha }k}\right)\left(\frac{1}{T_{\text{ref}}}-\frac{1}{T}\right)\right]\right)\exp\left(\frac{\text{qV}}{\text{AnKT}}\right)\left[\frac{\text{qV}}{\text{AnKT}}+1\right]$$

At MPP, $\frac{\text{dP}}{\text{dV}}=0$, therefore, solving for V recursively at MPP, equation (19) yielded equation (20).

(20) $$V_{\text{mpp}}=\frac{⍺\text{nKT}}{q}\left[\log_{e}\left(1+\frac{1\;}{1000\;\left(I_{\text{rs}}\;{\left[\frac{T}{T_{\text{ref}}}\right]}^{3}\;\exp\left[\left(\frac{qE_{\text{gap}}}{\alpha k}\right)\left(\frac{1}{T_{\text{ref}}}-\frac{1}{T}\right)\right]\right)}*\left[1+k_{i}\left(T-T_{\text{ref}}\right)\right]\frac{G}{G_{\text{ref}}}\right)-\left(\log_{e}\left(I_{\text{rs}}\;{\left[\frac{T}{T_{\text{ref}}}\right]}^{3}\;\exp\left[\left(\frac{qE_{\text{gap}}}{{\;}_{\alpha }k}\right)\left(\frac{1}{T_{\text{ref}}}-\frac{1}{T}\right)\right]\right)*\left(\frac{\text{qV}}{⍺\text{nKT}}+1\right)\right)\right]$$

To determine the current of the PV cell at maximum power point (I_mpp_), Substitute for V = V_mpp_ in equation (17) and automatically or ideally the value of I = I_mpp_ as expressed in equation (21). This simply means that when V =V_mpp_, I = I_mpp_.

(21) $$I_{\text{mpp}=}\;\left(\frac{⍺\text{nKT}}{qR_{s}}\;\log_{e}\left(1+\frac{{\;}_{1\;}}{1000\;\left(I_{\text{rs}}\;{\left[\frac{T}{T_{\text{ref}}}\right]}^{3}\;\exp\left[\left(\frac{qE_{\text{gap}}}{{\;}_{\alpha }k}\right)\left(\frac{1}{T_{\text{ref}}}-\frac{1}{T}\right)\right]\right)}\right)\;\left[1+k_{i}\left(T-T_{\text{ref}}\right)\right]\frac{G}{G_{\text{ref}}}\;\right)-\left(I_{\text{rs}}\;{\left[\frac{T}{T_{\text{ref}}}\right]}^{3}\;\exp\left[\left(\frac{qE_{\text{gap}}}{{\;}_{\alpha }k}\right)\left(\frac{1}{T_{\text{ref}}}-\frac{1}{T}\right)\right]\right)$$

Equations (20), (21) delineate the voltage and current at the maximum power point of a photovoltaic panel, respectively which will effectively compensate for panel losses.

To ascertain the ratio of output current to the output voltage of the PV cell, the differentiation of equation (11) with respect to the cell output voltage yields equation (22).

(22) $$\frac{\text{dI}}{\text{dV}}=-\frac{\;q\left(\;I_{\text{rs}}\;{\left[\frac{T}{T_{\text{ref}}}\right]}^{3}\;\exp\left[\left(\frac{qE_{\text{gap}}}{{\;}_{\alpha }k}\right)\left(\frac{1}{T_{\text{ref}}}-\frac{1}{T}\right)\right]\;\right)}{⍺\text{nKT}}\left(\exp\left(\frac{\text{qV}}{⍺\text{nKT}}\right)\;\right)$$

The developed technique is represented by equation (23), where **σ** is the Resultant conductance and $\frac{\text{dI}}{\text{dV}}\;\text{is}\;\text{the}\;\text{slope}$ measured in mho and A/V respectively.

(23) $$\sigma =\left(\frac{I_{\text{mpp}}}{V_{\text{mpp}}}-\frac{\text{dI}}{\text{dV}}\right)$$

Further substitution of equations (20), (21), (22) in equation (23) will yield equation (24).(24)
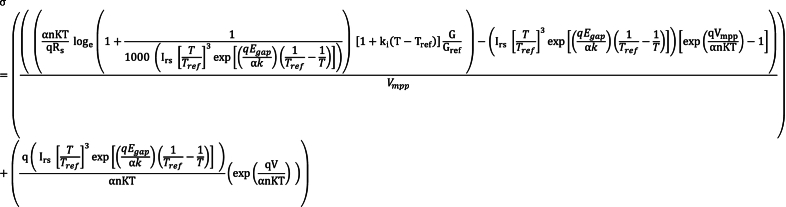

The recursive nature of equation (20) in this research contributes to the systematic, adaptive, automated, and compensative characteristics of the developed model. Its sequential, systematic, and automated design enables it to track the maximum power point efficiently by identifying the optimal operating point. This means that when the operating point shifts to the right, the model adjusts its tracking accordingly, and likewise when it shifts to the left, the model systematically readjusts without delay.

From equation (24), which represents the developed model, and equation (25), which denotes the fully substituted algorithm derived from the developed model, the conductance of the PV panel $\left(\frac{1}{Z_{\text{panel}}}\right)$ is represented by $\frac{\left(\;\left(\frac{⍺\text{nKT}}{qR_{s}}\;\log_{e}\left(1+\frac{{\;}_{1\;}}{1000\;\left(I_{\text{rs}}\;{\left[\frac{T}{T_{\text{ref}}}\right]}^{3}\;\exp\left[\left(\frac{qE_{\text{gap}}}{{\;}_{\alpha }k}\right)\left(\frac{1}{T_{\text{ref}}}-\frac{1}{T}\right)\right]\right)}\right)\;\left[1+k_{i}\left(T-T_{\text{ref}}\right)\right]\frac{G}{G_{\text{ref}}}\;\right)-\left(I_{\text{rs}}\;{\left[\frac{T}{T_{\text{ref}}}\right]}^{3}\;\exp\left[\left(\frac{qE_{\text{gap}}}{{\;}_{\alpha }k}\right)\left(\frac{1}{T_{\text{ref}}}-\frac{1}{T}\right)\right]\right)\left[{\;}_{\exp}\left(\frac{qV_{\text{mpp}}}{⍺\text{nKT}}\right)-1\right]\right)}{V_{\text{mpp}}}$ and conductance of the load $\left(\frac{1}{Z_{\text{load}}}\right)$ is represented by $-\frac{\;q\left(\;I_{\text{rs}}\;{\left[\frac{T}{T_{\text{ref}}}\right]}^{3}\;\exp\left[\left(\frac{qE_{\text{gap}}}{{\;}_{\alpha }k}\right)\left(\frac{1}{T_{\text{ref}}}-\frac{1}{T}\right)\right]\;\right)}{⍺\text{nKT}}\left(\exp\left(\frac{\text{qV}}{⍺\text{nKT}}\right)\;\right)$

From the developed model, for maximum power to be achieved, **σ** must be equal to or nearly zero, and therefore, $\frac{1}{Z_{\text{panel}}}=\frac{1}{Z_{\text{load}}}\text{.}$ The enhancement involved dynamically adjusting the impedance of the photovoltaic panel to match that of the load. A comparison between equations (1), (24) revealed significant differences. Equation (24) distinguishes itself from the validating equation (1) by its ability to formulate and model the Diode saturation current (Io) algorithmically, rather than assigning a constant value to it. Additionally, it incorporates the subtraction of load and panel differences, contrary to the addition approach in the existing model

Furthermore, of paramount importance in PV systems is the variable nature of Io, which is contingent upon cell temperature and necessitates adjustment accordingly [[49], [50], [51], [52]]. Another noteworthy variation is observed on the load side, where ($\frac{\text{dI}}{\text{dV}})$ now bears a negative sign after differentiation, in contrast to the positive sign in the current conventional OADC approach. Equation (21) introduces a constant value of -1 for Impp to strategically mitigate the impact of diode reverse saturation current during power delivery. Failure to account for this phenomenon resulted in substantial power loss. These enhancements collectively contribute to the markedly higher power output achieved through the new technique in comparison to conventional OADC methods.

### 2.3 Performance Metrics

A Measurement standard that is used to evaluate the performance of a model is known as a Performance metric. Ideal MPP Tracking Accuracy (IMTA) will be used to evaluate the effectiveness of the proposed model and it is obtained by taking the absolute difference of the average mean of new and old techniques, dividing it by the old technique, and taking the percentage as shown in equation (25) where N is the number of data points.

(25) $$\text{IMTA}=\frac{\left|\frac{1}{N}\sum P_{\text{old}}\right|-\left|\frac{1}{N}\sum P_{\text{new}}\right|}{\left|\frac{1}{N}\sum P_{\text{old}}\right|}\times 100$$

The performance of the proposed model was validated using optimized adaptive differential conductance [37]. The optimized adaptive difference conductance technique and Voltage-Control technique [53] were selected because of their good performance, low cost, and ease of implementation. Specifications of the input and output parameters used in the developed model were detailed as shown in Table 1.

**Table 1:** Input and output parameters.

INPUT DATA			OUTPUT DATA	
Names of Parameters	Symbol	Value	Names of Parameters	Symbol
Boltzmann's Constant	K	1.3805 × 10^−23^J/K	Current at Maximum Power Point	$I_{mpp}$
Diode Ideality Factor	A	1	Load Conductance (Slope)	$\frac{dI}{dV}$
Electron Charge	Q	1.6 × 10^−19^ C	Open Circuit Voltage	$V_{oc}$
Energy Band Gap	Ego	1.7622 × 10^−19^ J	Output Current	I
Number of cells	N	200	Output Power	P
Reference Temperature	Tref	298K	Panel Conductance	$\frac{I_{mpp}}{V_{mpp}}$
Reference Irradiance	G_ref_	1000W/m^2^	Photovoltaic Current	$I_{ph}$
Reverse Saturation Current	Irs	0.07A	Resultant Conductance	**σ**
Series resistance	R_s_	0.008Ω	Diode Saturation Current	Io
Cell Short Circuit Current Temperature Coefficient	k_i_	−0.0045/^o^C	Short Circuit Current	$I_{sc}$
Working Temperatures	T	250K, 298K, 350K,	Voltage Maximum Power Point	$V_{mpp}$
Voltage	V	0-V_oc_		
Working Irradiance	G	1000W/m^2^,700W/m^2^ 500W/m^2^		

## 3 Simulation results and discussions

The behavior of the developed model was simulated in a MATLAB environment using a mathematical equation (24) that characterizes the photovoltaic module. The model was explicitly tested for three classic PV parameters: the maximum power point tracking of a solar PV system enhancement, The effects of temperature and irradiance on the power generated and delivered to the load by the solar PV panel, and comparative comparison and validation of the developed IOADC with OADC and Voltage-Control techniques [53] in terms of the output power delivered to the load. These significant behaviors of solar PV modules were simulated using Equation (24), resulting in the data presented in Table 2, Table 3, Table 4.

**Table 2:** Resultant conductance (σ) variation with power, current and voltage at 1000W/m^2^ irradiance and temperature of 298K (STC).

1000W/M2			
S/N	P(W)	**σ** (mho)	VOLTAGE(V)
1	0.0000	0.40800	0.0000
2	21.7569	0.40080	2.1758
3	43.2684	0.38982	4.3516
4	64.3402	0.37305	6.5274
5	84.6415	0.34742	8.7032
6	103.6158	0.30828	10.8790
7	120.3289	0.24849	13.0548
8	133.2395	0.15715	15.2306
**9**	**139.8015**	**0.01764**	**17.4064**
10	135.8440	−0.1955	19.5822
11	114.5614	−0.5210	21.7580
12	64.89567	−1.0182	23.9338

**Table 3:** Current (A) variation with conductance, power and voltage at 1000W/m^2^ irradiance for different temperature.

S/N	250K			298K			350K			V(V)
	I (A)	**σ**(mho)	P (W)	I(A)	**σ**(mho)	P (W)	I (A)	**σ**(mho)	P (W)	
1	14.0507	0.5953	0.0000	10.0364	0.4080	0.0000	7.2720	0.2917	0.0000	0.0000
2	14.0236	0.5890	30.5125	9.9995	0.4008	21.7569	7.2228	0.2835	15.7153	2.1758
3	13.9786	0.5786	60.8292	9.9431	0.3898	43.2684	7.1521	0.2718	31.1231	4.3516
4	13.9040	0.5613	90.7572	9.8569	0.3730	64.3402	7.0508	0.2550	46.0232	6.5274
5	13.7805	0.5326	119.9346	9.7253	0.3474	84.6415	6.9054	0.2309	60.0992	8.7032
6	13.5759	0.4851	147.6917	9.5243	0.3083	103.6148	6.6969	0.1963	72.8559	10.8790
7	13.2367	0.4064	172.8029	9.2172	0.2485	120.3289	6.3979	0.1468	83.5232	13.0548
8	12.6748	0.2760	193.0451	8.7481	0.1572	133.2394	5.9690	0.0756	90.9110	15.2306
**9**	**11.7438**	**0.0599**	**204.4166**	**8.0316**	**0.0176**	**139.8015**	**5.3538**	**−0.0264**	**93.1895**	**17.4064**
10	10.2010	−0.2982	199.7587	6.9371	−0.1955	135.8440	4.4713	−0.17264	87.5583	19.5822
11	7.64482	−0.8914	166.3359	5.2653	−0.5210	114.5614	3.2056	−0.382464	69.7479	21.7580
12	3.40927	−1.8745	81.59683	2.7115	−1.0182	64.8957	1.3902	−0.6834	33.2724	23.9338

**Table 4:** Current (A) variation with conductance, power and voltage at 298k for different irradiance.

S/N	500W/m^2^			750W/m^2^			1000W/m^2^			V(V)
	I (A)	**σ** (mho)	P (W)	I(A)	**σ** (mho)	P (W)	I (A)	**σ** (mho)	P (W)	
1	5.4832	0.2362	0.0000	7.7598	0.3243	0.0000	10.0364	0.4080	0.0000	0.0000
2	5.4463	0.2290	11.8500	7.7229	0.3171	16.8035	9.9995	0.4008	21.7569	2.1758
3	5.3899	0.2180	23.4546	7.6665	0.3061	33.3615	9.9431	0.3898	43.2684	4.3516
4	5.3037	0.2012	34.6195	7.5803	0.2893	49.4799	9.8569	0.3730	64.3402	6.5274
5	5.1721	0.1756	45.0140	7.4487	0.2637	64.8278	9.7253	0.3474	84.6415	8.7032
6	4.9711	0.1365	54.0804	7.2477	0.2246	78.8476	9.5243	0.3083	103.6148	10.879
7	4.6640	0.0767	60.8875	6.9406	0.1648	90.6082	9.2172	0.2485	120.3288	13.0548
8	**4.1949**	**−0.0147**	**63.8911**	6.4715	0.0735	98.5652	8.7481	0.1572	133.2394	15.2306
**9**	3.4784	−0.15417	60.5464	**5.7550**	**−0.0661**	**100.1739**	**8.0316**	**0.0176**	**139.8015**	**17.4064**
10	2.3839	−0.367278	46.6820	4.6605	−0.2792	91.2630	6.9371	−0.1955	135.8440	19.5822
11	–	–	∗33.2462	2.9887	−0.6047	65.0270	5.2653	−0.5210	114.5614	21.7580

Table 2 is generated using the developed model in equation (24) at STC. It is pertinent to note from Table 2 that the maximum power point is identified at row nine, column two, while the minimum resultant conductance is noted at row nine, column three. This highlights the efficiency, efficacy, and accuracy of the developed algorithm, demonstrating alignment with the solar PV principle. According to this principle, the maximum power point of a PV panel occurs where power is highest and resultant conductance is lowest [1,[54], [55], [56], [57]].

To demonstrate the automatic and sequential compensating tracking mechanism of the developed model, it is important to observe that from rows 1–8 in column three, the algorithm tracks in a positive direction. This indicates that the operating point or maximum power point of the solar module is on the right-hand side. Conversely, from rows 10–12 in the same column, the algorithm starts re-tracking in a negative direction, suggesting that the maximum power point lies on the left-hand side. The positive region (Rows 1–8) signifies resultant conductance regions where the impedance of the PV module is smaller than the load impedance, while the negative region (Rows 10–12) indicates resultant conductance regions where the impedance of the PV panel exceeds the load impedance. At row nine is the point where the impedance of the PV module is equal to the impedance of the load and that's where the highest power and lowest impedance are recorded. These observations are consistent with the findings of Eze et al. [37], Haroun et al. [22], Teng et al. [58], and Li et al., [59]. This alignment with reputable scholars [22,37,58,59] confirms that the developed algorithm performs effectively, with an added improvement in power output.

**Fig. 4:**
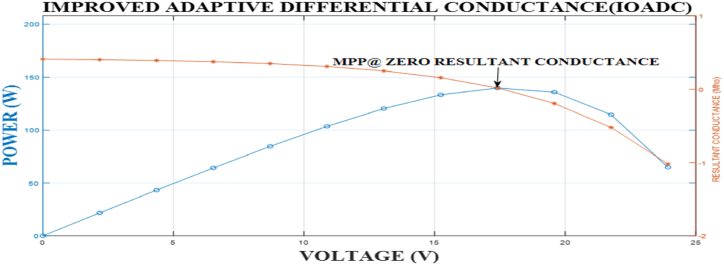
Developed model showing resultant conductance and power variation with voltage at STC

The relationship illustrated in Table 2 is graphically represented in Fig. 4. The graph indicates that as power increases, the resultant conductance decreases until it reaches a point known as the MPP, where the resultant conductance value approaches or is equal to zero. This demonstrates that as the output power increases, the output voltage also increases for V ≤ Vmpp. However, when V ≥ Vmpp, the output power decreases with an increase in voltage, and the resultant conductance inversely varies with the voltage in that region. Despite this, the resultant conductance remains inversely proportional to the voltage throughout the positive region. These findings align with the research conducted by Banakhr and Mosaad [27], Jalali Zand et al. [60], Sitbon et al. [61], and Kumar et al., [62].

Furthermore, the change in the sign of the resultant conductance immediately after reaching the MPP demonstrates the high accuracy and dynamic compensation tracking mechanism of the IOADC-developed model, as well as the model's adaptive nature when dynamically tracking the MPP. The ability of the developed model to locate the MPP very fast and accurately through a fast dynamic tracking mechanism makes it different from other algorithms.

Table 3 illustrates the impact of temperature on power, current, and conductance. The data indicates that the current maximum power points, resultant conductance, and maximum power points are all located in row nine, albeit in different columns, depending on the operating temperature.

From Tables 3 and it is evident that increasing temperature affects the solar PV conversion rate due to the squashed flat diode characteristics of silicon cells. Specifically, higher temperatures reduce the current and output power generated by the solar PV panel, while lower temperatures enhance the PV module output power. This behavior contrasts with the OADC technique and therefore forms part of the improvement. Additionally, the accuracy of the technique is confirmed, as the Maximum Power Point (MPP) corresponds to the lowest resultant conductance value. The observation that increasing temperature reduces the current and output power of the solar PV module, and vice versa, aligns with the analytical studies conducted by Arjyadhara and Chitralekha [12], Park et al. [53], Amelia et al. [63], Cuce et al. [48], and Ponnusamy & Desappan [64]. Despite agreeing with researcher [12,53,63] findings, IOADC power output was significantly enhanced.

**Fig. 5:**
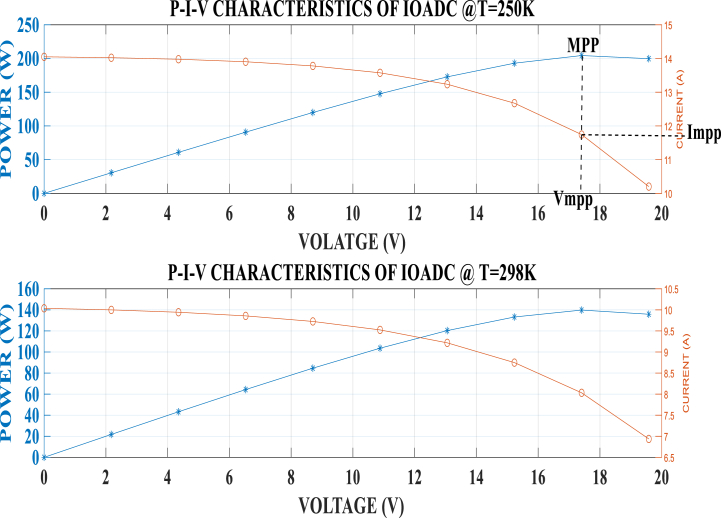
Power and current against voltage at temperature 250K and 298K

**Fig. 6:**
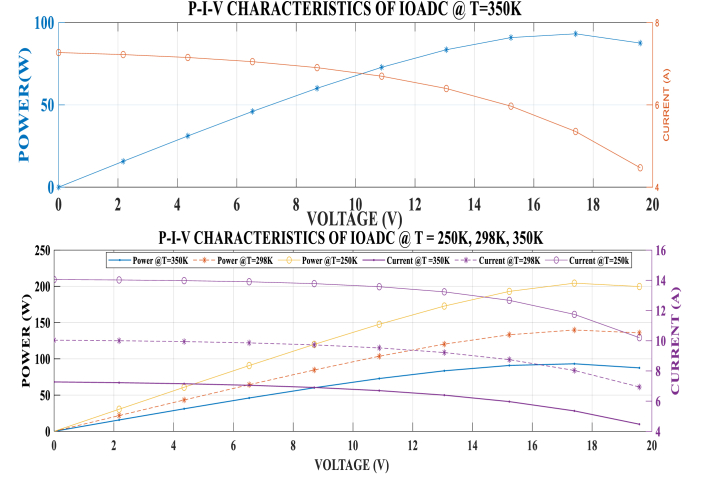
Power and current against voltage at different temperature.

From Fig. 5, Fig. 6, it is observed that as the current increases, the voltage decreases, and vice versa. Additionally, an increase in temperature results in a decrease in current and power, but an increase in voltage in the silicon SDM of a solar PV system. This drop in current and rise in voltage are due to the squashed flattened diode characteristic of silicon cells. This phenomenon occurs in silicon semiconductors because of the doping process involving phosphorus and boron, which creates regions with electron deficiency (p-type) and electron excess (n-type). The interface between these two layers generates free electrons (holes) [65,66].

From the presented results, it is evident that the MPP is located at the intersection of Vmpp and Impp along the P-V curve as indicated in Fig. 5. This intersection is clearly shown in the results, confirming that an increase in temperature decreases the power generated by the solar PV module and the quantity transferred to the load. This observation aligns with the findings of Sameh et al. [67], and Amelia et al. et al., [63]. It demonstrates that temperature significantly affects the performance of a PV panel, as it influences both the module saturation current and the photovoltaic current. This developed model showed a significant improvement by dynamically adjusting the saturation current characteristics to suit the environmental changes.

Table 4 shows the relationship between conductance, power, current, and voltage at a temperature of 298K and irradiance of 500W/m^2^, 750W/m^2,^ and 1000W/m^2^ respectively. It was noticed from the presented data that, the higher the irradiance the higher the current and power generated and transferred to the load. The higher the irradiance the higher the quantity of power transferred to the load because as the irradiance increases the tracking accuracy increases as illustrated in Table 4.

This finally showed that an increase in irradiance increases the quantity of power harnessed and transferred to the load from the solar PV module. The obtained results aligned with the research findings of Kollimalla et al. [17] and Traube et al. [68] on sudden changes in the irradiance of solar photovoltaics.

**Fig. 7:**
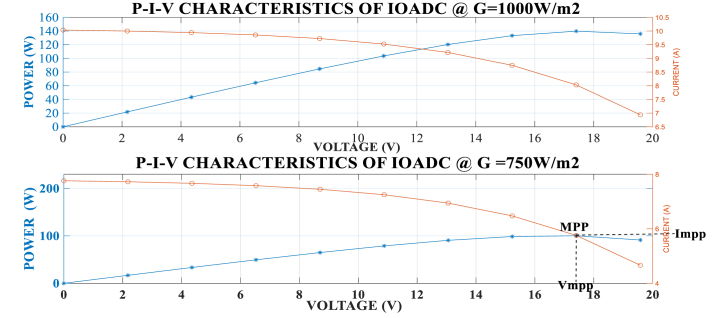
Power and Current against Voltage at different Irradiance 1000W/m^2^ and 750W/m.^2^.

**Fig. 8:**
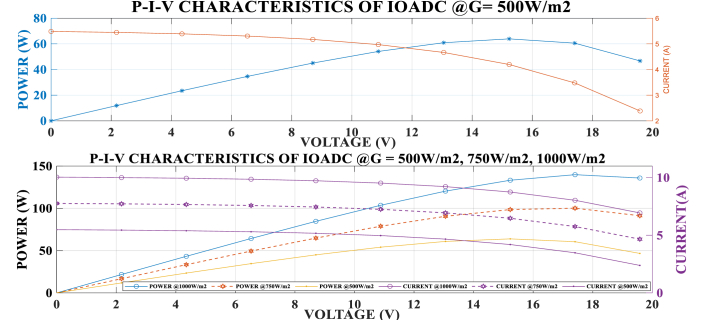
Power and Current against Voltage at different Irradiance.

Fig. 7, Fig. 8 illustrate the graphical relationship between power, current, and voltage at a temperature of 298K and irradiance levels of 500W/m^2^, 750W/m^2^, and 1000W/m^2^. The graphs indicate that power increases with higher irradiance and decreases with lower irradiance.

From the P-V-I graph, it is observed that power increases with voltage up to the Vmpp. Beyond this point, power starts to decrease even though voltage continues to rise until the Voc is attained. Similarly, power increases with a decrease in current until the Impp is attained. Beyond this point, power decreases as the current continues to decrease. The MPP is identified at the intersection of Impp and Vmpp on the P-V curve. These observations confirm that the developed model aligns with the characteristic behavior of solar PV models.

## 4 Discussion

Table 5 provides a comparative analysis of power output between IOADC and OADC techniques across various voltage levels (V) and irradiance conditions (500 W/m^2^ and 750 W/m^2^) at a temperature of 298 K. This analysis offers insights into the efficiency and effectiveness of these techniques in extracting power under varying atmospheric irradiance.

**Table 5:** Power validation characteristics at temperature of 298k

S/N	Improved Optimized Adaptive Differential Conductance (IOADC)		Optimized Adaptive Differential Conductance (OADC)		V(V)
	298 K				
	500W/m^2^	750W/m^2^	500W/m^2^	750W/m^2^	
POWER (W)					
1	0.0000	0.0000	0.0000	0.0000	0.0000
2	11.8500	16.8035	9.6743	14.6277	2.1758
3	23.4546	33.3615	19.1030	29.0099	4.3516
4	34.6195	49.4799	28.0921	42.9525	6.5274
5	45.0140	64.8278	36.3108	56.1246	8.7032
6	54.0804	78.8476	43.2014	67.9686	10.8790
7	60.8875	90.6082	47.8327	77.5534	13.0548
8	**63.8911**	98.5652	**48.6605**	**83.3346**	15.2306
9	60.5464	**100.1739**	43.1310	82.7675	**17.4064**
10	46.6820	91.2630	27.0998	71.6808	19.5822
11	33.0270	65.0270	∗10.1356	43.2690	21.7580

From Row 2 to Row 8, both IOADC and OADC demonstrate increasing power outputs with higher voltage levels, indicating a positive correlation between voltage and power generation. Particularly under lower irradiance (500 W/m^2^), IOADC consistently outperforms OADC in power output, with IOADC generating 11.8500W at 2.1758V compared to OADC's 9.6743W at the same voltage. This performance gap widens as IOADC achieves 63.8911 W at 15.2306V, surpassing OADC's 48.6605W, indicating IOADC's superior efficiency under low irradiance conditions.

Under higher irradiance (750W/m^2^), the performance difference is even more pronounced, with IOADC producing 33.3615W at 4.3516V compared to OADC's 29.0099W. This trend persists across all tested voltages, with IOADC peaking at 100.1739 W at 17.4064V, significantly higher than OADC's 83.3346W at 15.2306V Through comprehensive analysis and comparison, it has been determined that the utilization of IOADC results in a notable increase in the quantity of harnessed and transferred power, surpassing OADC by 20.21 %. This underscores IOADC's superior optimization for power harnessing, particularly at elevated irradiance levels.

Overall, IOADC consistently outperforms OADC in both low and high irradiance conditions, indicating superior optimization for power generation across a range of voltages. However, both techniques exhibit declining efficiency at higher voltage levels, suggesting the importance of optimal voltage regulation to maintain high power output. These findings are crucial for optimizing photovoltaic systems, where maximizing power output under varying environmental conditions is essential for efficiency and cost-effectiveness. Finally, the IOADC technique presents a notable advancement over OADC in terms of power output under different irradiance conditions, making it the preferred choice for applications requiring efficient power harnessing from solar energy.

**Fig. 9:**
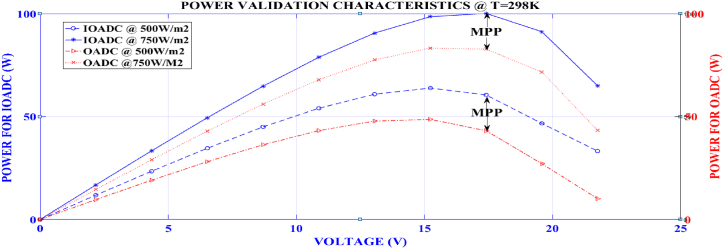
Plot of power against voltage for IOADC and OADC

Fig. 9 illustrates the graphical relationship between the power transferred using the Improved Optimized Adaptive Differential Conductance technique and the Optimized Adaptive Differential Conductance technique under varying atmospheric conditions. These tests were conducted with irradiance levels ranging from 500W/m^2^ to 750W/m^2^. The algorithm's performance was evaluated under two scenarios: varying irradiance with constant temperature and varying temperature with constant irradiance.

From the analysis of Fig. 9, it is evident that the IOADC technique consistently detected the Maximum Power Point more accurately under varying irradiance conditions, resulting in higher power output compared to the OADC technique. Specifically, at an irradiance level of 750W/m^2^, the power transferred to the load was 100.1739W for the IOADC technique and 83.3346W for the OADC technique. This demonstrates the superior performance of the IOADC technique under these conditions.

Further validation shows that the IOADC technique performs better than the OADC technique across all tested irradiance levels and temperatures. Based on the validation results, analysis, and evaluation, it can be concluded that the IOADC technique outperforms the OADC technique by an average of 20.21 % in terms of power transfer efficiency under varying atmospheric conditions. This performance enhancement is consistent with the expected trends in solar PV characteristics, confirming the effectiveness and reliability of the IOADC technique in maximizing power output under varying atmospheric conditions.

### 4.1 Real-world application of the developed MPPT technique

This section describes the implementation and testing of the newly developed IOADC technique in real-world conditions outside the simulation environment. This involves emulating the technique in actual solar energy systems to verify its effectiveness in optimizing power output under various atmospheric conditions, such as changes in sunlight, temperature, and shading. The goal is to demonstrate the practical effectiveness, reliability, and benefits of the IOADC technique in everyday real-life scenarios.

**Fig. 10:**
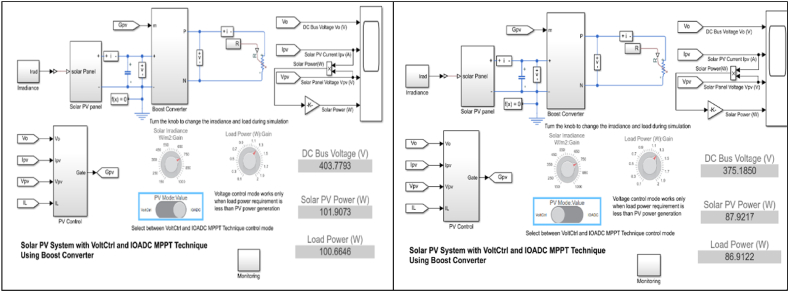
Real-world application of IOADC with voltage control MPPT at irradiance of 750W/m.^2^.

Fig. 10 illustrates the real-world application of IOADC and Voltage Control MPPT [53,69] at an irradiance level of 750W/m^2^. Although the real-world application can be validated under various atmospheric conditions (such as different irradiance levels and temperatures), an irradiance of 750W/m^2^ at a temperature of 298K was chosen for the validation process. The diagram clearly shows that the recorded values of IOADC closely align with those documented in the power validation characteristics presented in Table 5. This alignment demonstrates the IOADC model's efficient and effective performance in both simulations and real-world applications.

The similarity between the solar PV power and the power transferred to the load suggests that the IOADC model consistently transfers the generated power with minimal losses. Notably, the disparity between the generated power and the power transferred to the load remains consistent in the IOADC model, demonstrating its robust functionality across varying atmospheric conditions.

In contrast, the Voltage Control MPPT exhibits significantly lower power generation and power transfer to the load compared to the IOADC model. At an irradiance level of 750W/m^2^, the discrepancy between the generated power and the power transferred to the load for Voltage Control MPPT is measured at 13.7524W which is substantial.

This comparison highlights the superior performance of the IOADC model in terms of efficiency, reliability, and practical applications. The IOADC model ensures consistent power transfer while minimizing losses, making it a more effective solution for managing power generation and transfer in solar PV systems under varying atmospheric conditions.

**Fig. 11:**
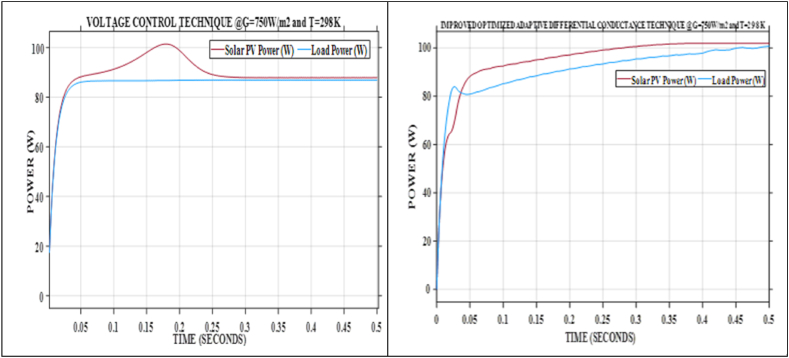
Improved optimized adaptive differential conductance technique and voltage control technique @ G = 750W/m^2^ and T = 298K

Fig. 11 illustrates the real-world application of power generation and transfer efficiency to the load when utilizing the IOADC technique compared to the Voltage Control (VC) technique under varying atmospheric conditions but the same testing conditions. Specifically, at an irradiance of 750W/m^2^, the IOADC technique transfers 100.6646W to the load, outperforming the VC technique, which achieves 86.9122W. This indicates a notable improvement of 15.8233 % in power transfer efficiency with the IOADC technique.

The superiority of the IOADC technique is consistently demonstrated across various irradiance and temperature levels. Comprehensive validation results, analytical insights, and calculations derived from equation (25) confirm the enhanced performance of the IOADC technique. This robust validation across diverse environmental conditions reinforces the reliability and effectiveness of the IOADC technique compared to the VC technique.

Furthermore, the performance of the IOADC technique aligns closely with the characteristic trends of solar PV systems. The validation graph in Fig. 11 not only substantiates the superiority of the IOADC technique across different environmental conditions but also demonstrates its conformity to the expected power generation and load transfer curves of solar PV systems. This visual representation distinctly illustrates how both techniques follow these characteristic trends, further emphasizing the efficacy and reliability of the IOADC technique in comparison to the VC technique. The comprehensive validation and graphical evidence firmly establish the IOADC technique as a superior method for power transfer in solar PV systems.

### 4.2 Performance of the IOADC under varying atmospheric conditions

The analysis presented in Table 3, Table 4, Table 5 and Fig. 5, Fig. 6, Fig. 7, Fig. 8, Fig. 9 provides valuable insights into the efficiency and effectiveness of these techniques for extracting power under varying atmospheric conditions. Additionally, the performance of the developed IOADC algorithm was evaluated under varying atmospheric temperatures (250K, 298K, 350K) and irradiance levels (500W/m^2^, 750W/m^2^, 1000W/m^2^) and the results demonstrated a significant improvement in output power and tracking accuracy. Table 5 and Fig. 9 highlight the simulation results for tracking accuracy and improved output power of the IOADC compared to the OADC. The findings indicate that under rapidly changing atmospheric conditions, both algorithms accurately track the MPP, but the IOADC shows superior output power performance. This suggests that the OADC experiences some power loss in such conditions. Hence, the developed model significantly enhances power output and MPPT tracking accuracy under varying atmospheric conditions compared to the OADC. Furthermore, the IOADC demonstrates practical improvements in power output when compared to VC, highlighting the superiority and universality of the developed model.

### 4.3 Summary of the findings

The Main Findings/Justifications of this Research are summarized as follows.

• The validation of the developed IOADC technique with Eze et al. [37] demonstrated a notable 20.21 % improvement in power harnessing and transfer from solar PV modules to the load. This substantial enhancement underscores the effectiveness of the proposed MPPT methodology.
• Real-world application of the developed technique, utilizing the voltage control method from Park et al. [53], also yielded positive results, showcasing a 15.82 % improvement in power harnessing and transfer efficiency under varying atmospheric conditions. These findings provide alternative insights into practical applications for enhancing solar PV system performance.
• The research established a direct correlation between temperature variations and power output, revealing that increased temperature negatively impacts both power harnessing and power transfer to the load under varying atmospheric conditions. Conversely, the study confirms that higher irradiance levels positively correlate with enhanced power generation and delivery, consistent with the findings of Kollimalla et al. [17] and Traube et al. [68] but with an improved power output that shows the efficiency of the new model.
• The proposed model demonstrated exceptional robustness in tracking the MPP of the solar PV system, even under varying atmospheric conditions. This resilience contributes to the reliability of the IOADC MPPT technique in real-world scenarios making it one of the exceptional non-intelligent MPPT techniques.

### 4.4 Recommendations/Future studies

• Future research should explore the scalability and adaptability of this technique in larger PV installations.
• Additionally, this research methodology can be replicated in intelligent MPPT techniques.
• Temperature sensors can be used to replace the dynamic saturation current adjustment path

## 5 Conclusion

The Improved Optimized Adaptive Differential Conductance (IOADC) Technique was successfully developed, simulated, and analyzed using MATLAB software. The results demonstrated that the developed model is effective, efficient, accurate, and robust. Specifically, at low temperatures and high irradiance, the IOADC model transfers the highest generated power to the load, thereby reducing recharge duration. The output of the developed maximum power point tracking model showed a systematic and sequential matching of the impedance of the PV panel with the impedance of the load under varying irradiance and temperature. Additionally, the effect of module saturation current on the output current of the solar PV panels was improved by incorporating the dynamic saturation current adjustment in the model. Compared to existing models, the developed model outperformed the voltage control and OADC models by 15.82 % and 20.21 % respectively, in terms of real-world application and simulation power transferred to the load. Furthermore, this research highlighted not only the enhancement of power transfer to the load at varying irradiance and temperature but also the impact of module saturation current and temperature on the output power. The developed model has significant potential for application in MPPT-based fast-charging electric vehicle stations. Upon commercialization, it will minimize energy loss and maximize the effective utilization of solar PV-generated energy, aligning with the goals of PV designers and engineers.

## CRediT authorship contribution statement

**Val Hyginus Udoka Eze:** Writing – original draft, Methodology, Conceptualization. **Martin Chinweokwu Eze:** Validation, Supervision. **Samuel A. Ugwu:** Writing – review & editing, Software. **Valentine S. Enyi:** Writing – review & editing, Visualization. **Wisdom O. Okafor:** Writing – review & editing. **Chibuzo C. Ogbonna:** Writing – review & editing, Conceptualization. **Ogbonna U. Oparaku:** Supervision.

## Data and code availability statement

The datasets generated during and/or analyzed during the current study are available from the corresponding author upon reasonable request. The data have been deposited at https://codeocean.com/capsule/2987328/tree and can be easily accessed using the provided link.

## Ethical declaration

Review and/or approval by an ethics committee was not needed for this study because it contains no human sample or subject.

## Declaration of competing interest

The authors declare that they have no known competing financial interests or personal relationships that could have appeared to influence the work reported in this paper.
